# *Toxoplasma gondii* Prevalence, United States

**DOI:** 10.3201/eid1304.061355

**Published:** 2007-04

**Authors:** Jeffrey L. Jones, Deanna Kruszon-Moran, Marianna Wilson

**Affiliations:** * Centers for Disease Control and Prevention, Atlanta, Georgia, USA; † Centers for Disease Control and Prevention, Hyattsville, Maryland, USA

**Keywords:** Toxoplasma, toxoplasmosis, prevalence, serology, letter

**To the Editor:** We correct the prevalence of *Toxoplasma gondii* immunoglobulin (Ig) G antibodies published in Emerging Infectious Diseases in 2003 (*1*). An incorrect cutoff value in the computer program used to calculate seropositivity of anti–*T. gondii* IgG antibody resulted in some incorrect prevalence rates. We discovered this error when analyzing more recent National Health and Nutrition Examination Survey (NHANES) data.

The cutoff value for anti–*T. gondii* IgG seropositivity used in the prior publication (*1*) was >6 IU, which is the correct value for NHANES III 1988–1994 data (*2*) but not for NHANES 1999–2000 data. Because of a change by the *T. gondii* test kit manufacturer, the cutoff value for NHANES 1999–2000 seropositivity data was increased to ≥10 IU. This cutoff change from >6 to ≥10 IU does not cause a large difference in the *T. gondii* seroprevalence reported. In addition, it does not change the overall findings of the article or the overall relationship between NHANES III (1988–1994) and NHANES 1999–2000. However, it does produce a borderline change for 2 demographic subgroups (non-Hispanic white persons and persons born in the United States), for whom the difference from NHANES III to NHANES 1999–2000 data reached statistical significance at p<0.05 in the *t* test, but the 95% confidence intervals (CIs) for the prevalence estimates for these groups still overlapped between NHANES III and NHANES 1999–2000 (i.e., the *t* test is a less conservative measure of association than CI).

After this correction, the overall *T. gondii* antibody prevalence according to NHANES 1999–2000 data changed from 15.8% (95% CI 13.5%–18.1%) to 14.3% (95% CI 12.3%–16.2%). The Table shows the overall and stratified seroprevalence rates for NHANES 1999–2000 (corrected) compared with NHANES III (no corrections needed).

**Table:** 1Comparison of*Toxoplasma gondii* IgG antibody seroprevalence, NHANES 1999–2000 and NHANES III (1988–1994)*†

	NHANES 1999–2000			NHANES III (1988–1994)		
	N‡	Prevalence	95% CI	N‡	Prevalence	95% CI
Total	4,234	14.3	12.3–16.2	11,132	16.0	14.5–17.5
Sex						
Male	2,013	15.2	12.4–18.0	5,144	16.7	14.8–18.6
Female	2,221	13.4	11.2–15.5	5,988	15.3	13.5–17.0
Race/ethnicity						
Non-Hispanic white	1,293	10.8	8.1–13.6	3,304	14.3	12.5–16.2
Non-Hispanic black	1,027	16.8	13.4–20.3	3,674	18.0	16.1–19.8
Mexican American	1,553	14.2	10.1–18.4	3,661	18.3	16.7–20.0
Age group, y						
12–19	2,105	7.3	4.7–10.0	2,749	8.5	6.4–10.5
20–29	735	11.9	9.5–14.4	3,100	15.2	12.1–18.3
30–39	726	17.0	12.9–21.2	2,960	16.1	14.6–17.6
40–49	668	18.7	15.0–22.3	2,323	22.2	19.4–25.0
Country of birth						
United States	3,211	10.5	8.3–12.8	8,606	14.1	12.7–15.5
Not United States	995	32.0	24.0–39.9	2,493	27.9	24.1–31.7

*IgG^,^ immunoglobulin G; NHANES, National Health and Examination Survey; CI, confidence interval. †Sex, race/ethnicity, and total values are age-adjusted to the 2000 census–estimated population using the 4 age categories shown. ‡Totals for the race/ethnicity and country-of-birth categories do not add up to the total number because of an “other” category (not shown) or because persons did not provide a response to country-of-birth questions.
